# Seasonal Snowpack Classification Based on Physical Properties Using Near-Infrared Proximal Hyperspectral Data

**DOI:** 10.3390/s21165259

**Published:** 2021-08-04

**Authors:** Mohamed Karim El Oufir, Karem Chokmani, Anas El Alem, Hachem Agili, Monique Bernier

**Affiliations:** INRS-Institut National de la Recherche Scientifique (INRS), Québec, QC G1K 9A9, Canada; karem.chokmani@ete.inrs.ca (K.C.); anas.el_alem@ete.inrs.ca (A.E.A.); hachem.agili@ete.inrs.ca (H.A.); monique.bernier@ete.inrs.ca (M.B.)

**Keywords:** seasonal snowpack, metamorphosis, classification, hyperspectral imaging, near-infrared

## Abstract

This paper proposes an innovative method for classifying the physical properties of the seasonal snowpack using near-infrared (NIR) hyperspectral imagery to discriminate the optical classes of snow at different degrees of metamorphosis. This imaging system leads to fast and non-invasive assessment of snow properties. Indeed, the spectral similarity of two samples indicates the similarity of their chemical composition and physical characteristics. This can be used to distinguish, without a priori recognition, between different classes of snow solely based on spectral information. A multivariate data analysis approach was used to validate this hypothesis. A principal component analysis (PCA) was first applied to the NIR spectral data to analyze field data distribution and to select the spectral range to be exploited in the classification. Next, an unsupervised classification was performed on the NIR spectral data to select the number of classes. Finally, a confusion matrix was calculated to evaluate the accuracy of the classification. The results allowed us to distinguish three snow classes of typical shape and size (weakly, moderately, and strongly metamorphosed snow). The evaluation of the proposed approach showed that it is possible to classify snow with a success rate of 85% and a kappa index of 0.75. This illustrates the potential of NIR hyperspectral imagery to distinguish between three snow classes with satisfactory success rates. This work will open new perspectives for the modelling of physical parameters of snow using spectral data.

## 1. Introduction

Snow is one of the most important components of the cryosphere [1]. It significantly contributes to the water balance, climate, and economy of many geographic regions [2,3,4]. The snow cover, also called snowpack, is the set of snow layers that pile up on the ground throughout winter [5]. This snow cover is highly variable due to the complex distribution of precipitation, wind, and radiation after snow deposition [6]. Each layer is separated from the previous one by a superposition plane. This interface between two layers is of unequal granulometry and density, and forms a strong link between the layers by resisting shear stress [5].

The snow on the ground undergoes a continuous transformation process, known as metamorphism, that controls the physical properties of snow [7,8]. Indeed, snow grain size and snow density, which are used to define snow grain types, change over time due to different metamorphic mechanisms [9,10,11]. This continuous variation in the physical properties of snow along the vertical structure of the snowpack allows scientists to objectively characterize the metamorphic state of snow [5,12,13].

The terms used to describe the spatial and temporal evolution of snow are important for all aspects of snow studies and applications [14]. Indeed, a simple description is sufficient to characterize the vertical structure of snowpack. That is why, in the early research on snow cover, it was necessary to classify the seasonal snowpack according to its physical properties. In the 1970s, Pahaut [15] noted that snow is compacted over time and proposed to classify densities based on the type of snow. In response to the growing need for standardization, an international classification system was subsequently proposed by Colbeck [16] and later revised by Fierz [5] in their book entitled “International Classification for Seasonal Snow on the Ground.” This classification system based on visual recognition and related techniques has since then been used by snow physics practitioners to classify snow on the ground [17].

Despite the improvements in the measurement of the physical properties of snow, the classification of snow on the ground remains one of the most challenging issues. Indeed, the classification of measured data on the vertical profile of snowpack is a laudable objective for several reasons [4]. In economic and social terms, permission for many winter recreational activities depends on knowledge of the vertical structure of the snowpack, which makes this task paramount and valuable [18]. Also, meteorological conditions and solid precipitation data remain insufficient to predict avalanche risks [6]. To do so, it is necessary to document the internal evolution of the snowpack, hence the need for frequent and regular observations and measurements [6]. These measurement techniques are often based on direct observations using a magnifying glass to classify granulometry stratigraphy and snow grain shape, and a snow sampler to measure density. Such direct measurements makes it possible to improve existing snow cover models which are used to quantitatively simulate changes over time for hydrological applications such as the snow water equivalent [4]. However, these classification techniques require the use of in situ sampling for hydrological and avalanche studies. Previous experimental results have shown that traditional methods used for the classification of snow samples have undeniable weaknesses (laborious methods requiring several hours of work) [19]. These methods rely on manual measurements of the physical characteristics of snow and are not suitable for the evaluation of individual samples in large-scale, fast-paced production [20,21]. Also, because of the sensitivity of snow grain morphology to changes in local temperature and humidity, field measurements generally contain significant errors [22,23]. Therefore, a fast, objective, and non-invasive classification technique of the physical properties of snow is long overdue.

Hyperspectral imaging is a new technique based on spectroscopic analyses. It is fast, non-destructive, facilitates real-time measurements, and is widely used in various fields, namely agriculture [24,25], pharmacy [26], and the food industry [27,28]. It has proven its effectiveness in field, laboratory, and industrial applications [29,30]. Indeed, it has already been demonstrated that the near infrared (NIR) spectrum is sensitive to the physical parameters of snow [31,32,33,34]. In fact, snow granulometry is clearly visible in the NIR and the short waves of infrared regions (SWIR) [35,36]. Eppanapelli et al. [37] found that the spectral reflectance of snow in the NIR spectrum is inversely proportional to the liquid water content of the snow. In addition, the absorption of ice in the NIR spectrum is very high [38], so the effect of impurities such as mineral dust and soot is negligible beyond wavelenths of 1000 nm.

The above results highlight the potential of NIR hyperspectral data to gather information on the physical properties of snow. In fact, many studies and pieces of research have been done for this type of application, but they have treated each parameter individually. For example, snow grain size has been correlated with reflectance of small discrete snow samples using either the integrating sphere method (e.g., DUFISSS [39], IceCube [40], and Infrasnow [41]) or a high spectral resolution NIR photography method [42,43]. Similarly, the liquid water content of the snow was modeled based on the reflective properties of the snow. The results show that liquid water content is inversely correlated with NIR reflectance [33,37]. Density was also correlated with this part of the spectrum. Bohren and Beschta [44] found that the correlation between spectral reflectance and snow density is probably the result of other changes that occur in the aging process. They concluded that there is a relationship between this parameter and reflectance. As stated, most previous studies have focused on relating a single physical property of snow (density, grain size, or liquid water content) to reflectance. Reflectance-based modeling of their combined effect is not yet known.

This research aims at assessing the potential of hyperspectral imaging in the NIR spectrum for the classification of homogeneous layers of the vertical profile of snowpacks in terms of density, grain size, and snow grain shape. An exercise comparing field data and the corresponding spectral data was also carried out for evaluation purposes.

## 2. Materials and Methods

### 2.1. In Situ Measurements

**Figure 1:**
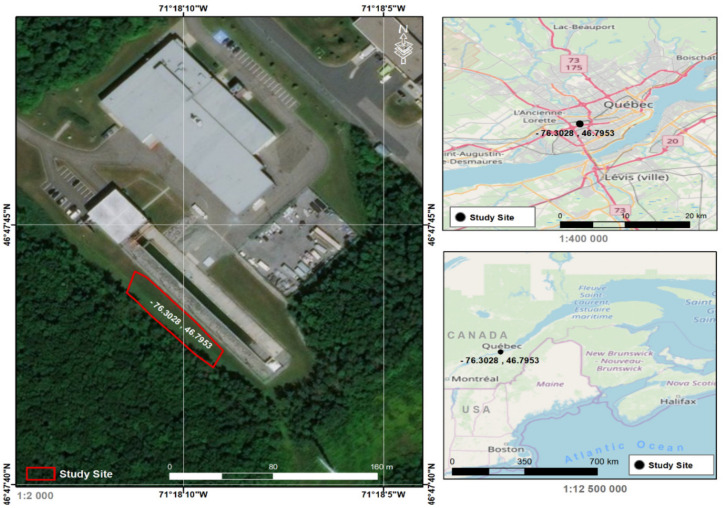
Geographical location of the sampling site.

In order to be able to carry out this study, two types of data had to be collected: the physical properties of the snowpack and its optical properties. The same equipment and sampling techniques were used throughout the study. Weekly physical and optical measurements of the snow pit were carried out on land owned by the National Institute for Scientific Research (INRS) in the City of Québec (Figure 1). Measurements were taken between 08 h00 and 12 h00 on sunny days without wind. The City of Québec is characterized by winter temperatures ranging between −10 °C and −25 °C, with a significant accumulation of snow. The snowpack in this region is dry from January to mid-February due to low temperatures and becomes wet in March as the temperature rises. An open area of approximately 20 m × 5 m was selected as the study site. Major roughness characteristics and slopes were avoided by sampling only smooth and flat surfaces. Surfaces containing impurities were also avoided in order to retrieve as much representative information on the seasonal snowpack as possible. The sampled snow depth ranged between 54 cm and 105 cm.

#### 2.1.1. Physical Properties of Snow

**Figure 2:**
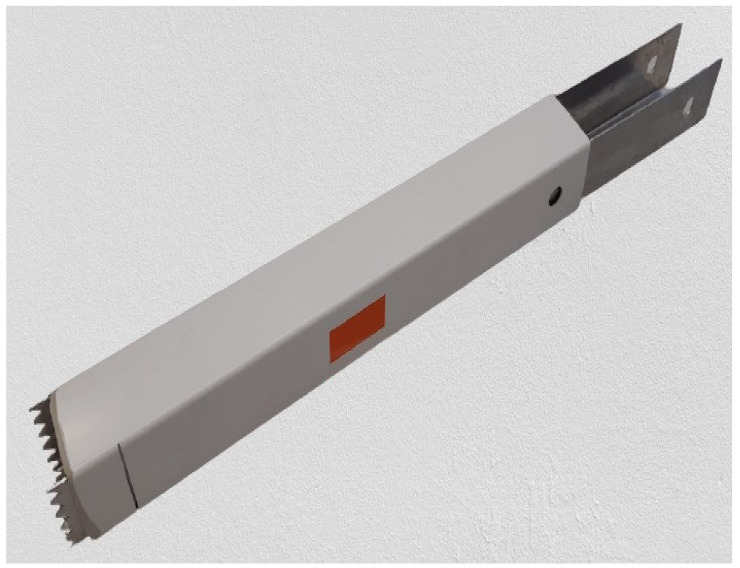
Snow core sampler.

**Figure 3:**
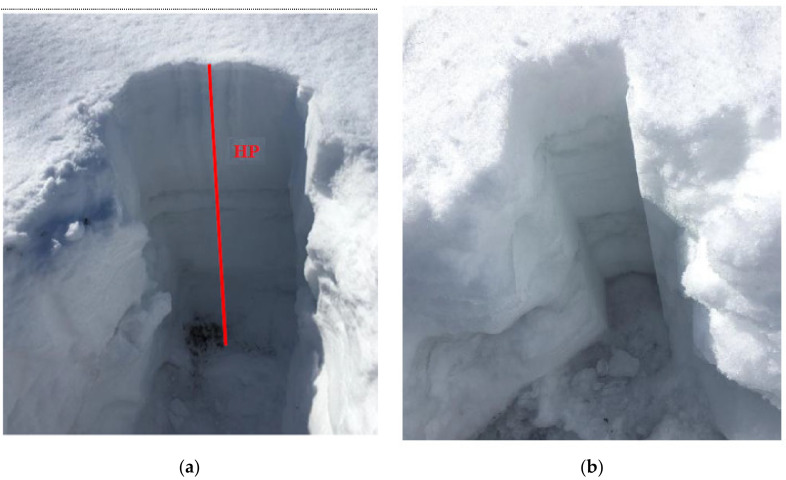
(**a**) Snow pit, HP—height of the pit; (**b**) Snow pit after profile extraction, winter 2018.

**Figure 4:**
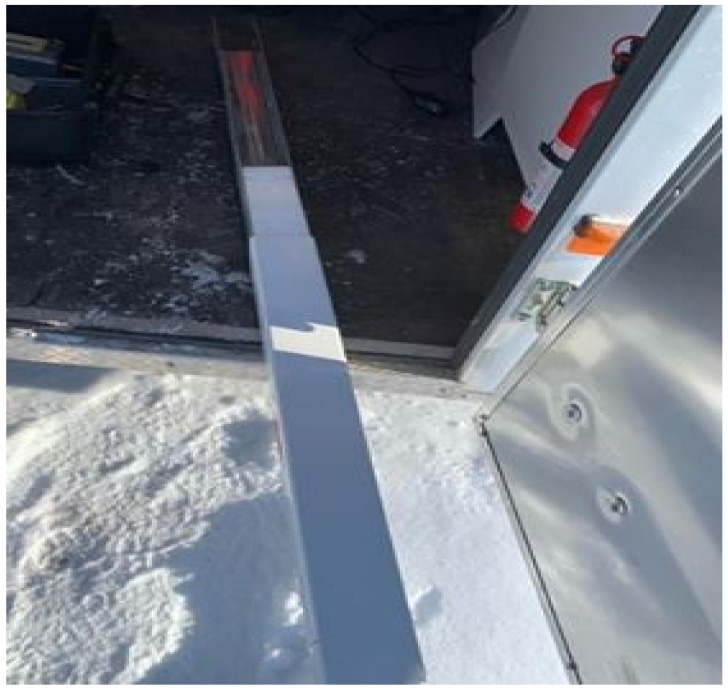
The vertical profile within the snow corer, winter 2018.

Figure 2 shows the portable rectangular snow core sampler used to sample the snowpack (custom-built). This instrument was used to extract snow cores and retrieve the vertical profile of the snowpack, allowing a visual assessment of the vertical stratigraphy of the snow cover. Both the physical (density, grain sizes, and grain types) and optical (spectral reflectance) properties of snow were assessed on the snow core. The snow corer is composed of a metallic inner component and a plastic cover with a triangular sawtooth cutting part at the end. The corer allowed us to measure the height (cm) of the profile and to differentiate the homogeneous layers composing the vertical profile. The dimensions of the core sampler (10 × 10 × 120 cm) allow the entire vertical profile of the snowpack to be recuperated and the snow to be retained. The first step in collecting a snow sample was to dig out the snow pit (Figure 3a). To do this, the snow sampler was inserted through the surface of the snow (Figure 3b) so as to extract both the surface snow and the deeper layers of snow. The physical and optical properties of the snow, as well as the identification of the snow layers and their thickness, were measured directly on the core. The core sampler is graduated with marks indicating the measurement scale (centimeters); this allowed us to measure the thickness and position of homogeneous layers of snow. The vertical profile was extracted by pushing the core through the snowpack until the serrated cutting end reached the ground surface. Finally, the sampler was carefully removed and placed horizontally on the snowpack to measure the physical characteristics (Figure 4).

#### 2.1.2. Optical Properties of Snow

**Figure 5:**
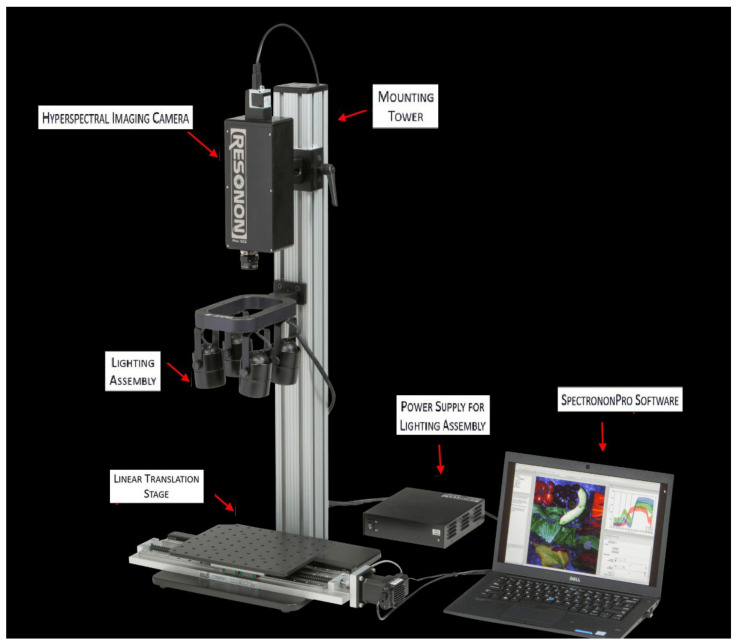
Proximal hyperspectral image acquisition device.

Figure 5 shows the Pika NIR hyperspectral imaging line-scanning camera (RESONON Company) used to measure the optical properties of the snowpack. It measures the reflected light at NIR wavelengths ranging from 900 nm to 1700 nm. Reflectance spectra were calculated by measuring the radiance reflected by the snow surface and the radiance reflected by a reference target (Spectralon 99%), with the latter having near-Lambertian reflection properties when viewed from the nadir. In addition to the camera, the device is equipped with a mobile platform (metal bench) of linear translation stage, allowing rapid image acquisition; a mounting tower; a set of four halogen lights (500 watts each), placed perpendicular (90° ± 5°) to the snow sample for equal illumination; and Spectronon Pro (Resonon Inc., Bozeman MT, USA) software, dedicated to the acquisition and analysis of hyperspectral data.

**Figure 6:**
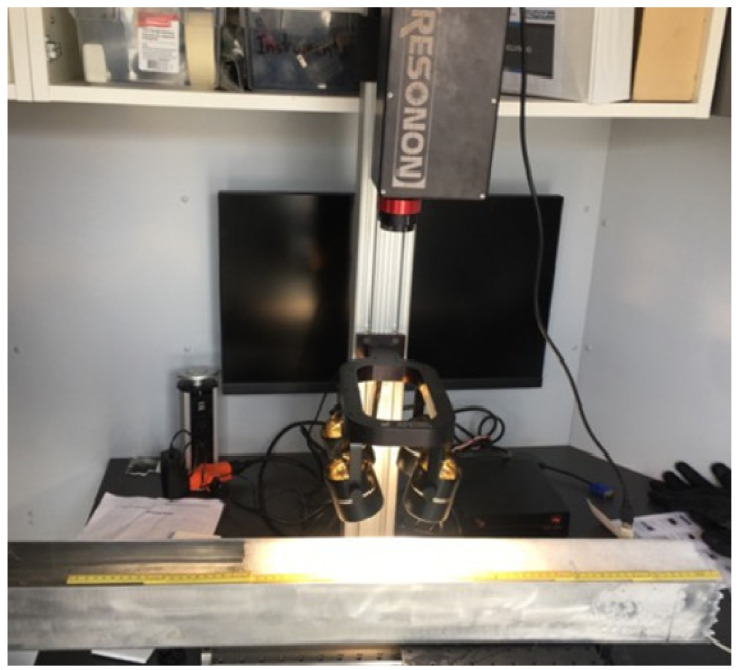
Hyperspectral acquisition of the snow samples’ vertical profile.

The optical properties were measured by placing the snow core horizontally on the mobile platform relative to the camera’s field of view (nadir) to be scanned line by line (Figure 6). The speed of the platform was adjusted for the spatial resolution of the vertical and horizontal axes to be equal, to avoid any size distortion and to fit with the predetermined exposure time of the camera. Using the proprietary Resonon Spectronon software, the snow core was scanned in a short time of 5 s. Hyperspectral images were created by gathering the images line by line while the sample was being interpreted relative to the camera. Hyperspectral images were acquired in a 3D form called hyper-cube, composed of two spatial dimensions (x, y) and one spectral dimension (λ). Each hyperspectral cube consists of 148 spectral in-formation points at a spectral resolution of 5.5 nm. Each pixel in the hyperspectral image contains the spectrum of that specific position characterizing the physical properties within that pixel.

### 2.2. Statistical Analysis of Data

Hyperspectral data are characterized by their high dimensionality and collinearity. A statistical approach is therefore needed to extract useful information from the NIR spectrum. Dimensionality reduction methods have the advantage of being fit to process spectral data by reducing their high dimensionality while minimizing the loss of useful information. In the present study, a principal component analysis (PCA) was used for data visualization and spectral dimensionality reduction. A PCA-based hierarchical ascending classification (HAC) was used thereafter to classify the reflectance into different groups according to snow type, grain size, and density.

#### 2.2.1. Principal Component Analysis (PCA) of NIR Spectra

Due to the complexity of high dimensionality problems, several dimensional reduction methods are suggested in the literature, such as the PCA [45]. This technique is used to interpret spectral data by identifying the most important directions of variability in the multivariate data space and expressing the spectral data in such a way as to highlight their similarities and differences. The PCA is a mathematical procedure that decomposes spectral data into several main components which are linear combinations of the original spectral data and can represent the spectral variations common to all spectral data.

The PCA is the best spectral band selection method for dimensionality reduction without the loss of useful information [46]. In this paper, the principal component coefficients, also known as charges, were investigated for their usefulness in reducing the dimensionality of hyperspectral data. Thus, the factor maps could be interpreted on the principal planes, making it possible to differentiate snow classes on the basis of their spectrum. To do this, a PCA was applied to all spectral data corresponding to the homogeneous snow layers extracted from the hyperspectral images (from 900 nm to 1700 nm). The first principal components (PC) resulting from the PCA are generally used to examine the common characteristics of the samples and their clustering. Thus, samples having similar spectral signatures tend to aggregate in the score diagram of the first two or three PCs.

#### 2.2.2. Hierarchical Ascending Classification of Near Infrared Spectra

The hyperspectral data matrix obtained after the PCA was studied using the hierarchical ascending classification (HAC) [47,48]. It is a powerful unsupervised classification method that allows scientists to build tree structures from data similarities and to see how different subclasses are related to each other [49]. The HAC analyzes the distance between points of the multidimensional space using progressive clustering and results in the representation of the two closest successive points in the form of a classification tree. To do this, data points are grouped into n subclasses by testing their relative proximity. Successive classes are grouped in the same way into n-1, n-2, n-3, etc. classes, until one single class is reached. Each given class is included in the next class, and the set of these classes is presented in the form of a classification tree. The level of aggregation is given by the ordinate axis with a relative scale from 0% to 100%. The abscissa axis only represents a qualitative order according to the progressive increase of the aggregation criterion of the main subsets.

The aim of this classification method is to group data into classes by assuming the existence of similarities between the different points, using only hyperspectral data, and without prior knowledge. The spectrum is therefore used to classify snow samples according to their similarity in terms of metamorphosis degree (weakly, moderately, and strongly metamorphosed snow), and consequently to obtain homogeneous optical classes.

### 2.3. Accuracy Assessment

**Figure 7:**
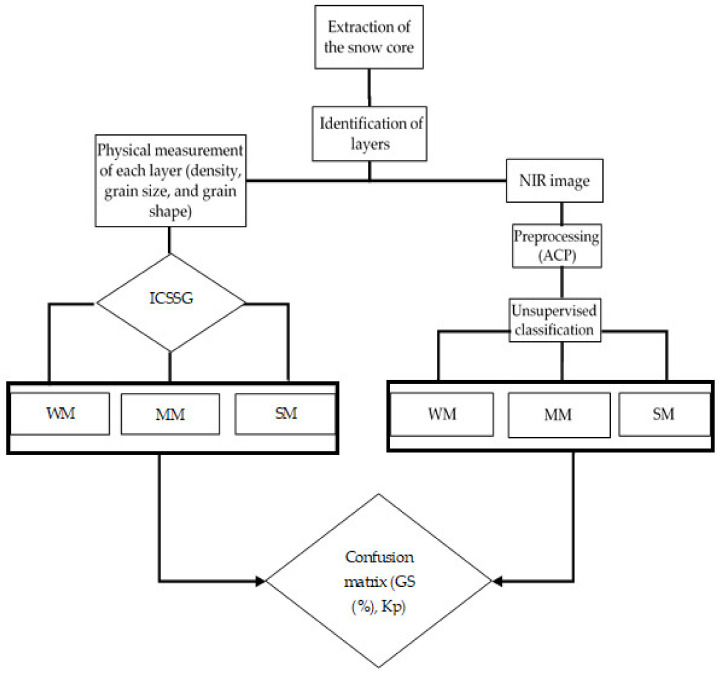
Flowchart of the methodological approach. WM—weakly metamorphosed; MM—moderately metamorphosed; SM—strongly metamorphosed; GS—global success; Kp—kappa index; ICSSG—International Classification for Seasonal Snow on the Ground.

To evaluate the performance of the classification, a confusion matrix was analyzed [50]. It consists of comparing measured classes (physical measures) with estimated classes (optical measures) and quantifying the precision of the approach used by calculating success rates, errors (commission and omission), and precision indices, namely the kappa index [50]. The latter permits us to quantify the level of agreement between the classes of the physical characteristics measured and those estimated. Concordance is considered to be good for kappa values greater than 0.6 and excellent for values above 0.8. The flowchart in Figure 7 illustrates the methodological approach adopted.

## 3. Results and Discussion

### 3.1. Descriptive Analysis

Each layer of the snowpack has its own history, which can be very different from that of its upper or lower layers and results in very different evolution patterns [51]. Thus, all the different values and dimensions of physical properties can coexist simultaneously on the same vertical axis of the snowpack. In this regard, it is necessary to classify the physical properties of each snow layer. In a snowpack, the density, type, and size of grains in each snow layer usually increase with depth, but exceptions are very frequent and generally correspond to instabilities [52]. In this work, in situ sampling of the physical properties of snow was inspired by the work of Pahaut [15], and the snowpack was treated as a succession of layers. Twenty-four snow cores were collected for this study (from 19 January to 27 March in 2018, from 10 January to 3 April in 2019, and from 29 January to 9 March in 2020). Indeed, we collected weekly data during three winter seasons, in order to obtain data as diverse as possible and to guarantee a wide range of degree of metamorphism. Table 1 and Table 2 show reports of data for temperature and snow accumulation on the ground for the winters of 2018, 2019, and 2020 according to https://climate.weather.gc.ca/, accessed on 15 July 2021. For each snow profile, a series of physical and optical measurements were made and recorded. A mobile laboratory exposed near the sampling site that keeps the same degree of outside temperature allowed us to take physical and optical data within approximately 10 min. Snow layers were visually identified by observing the changes in the size and type of grains on the core’s surface. For each identified layer, the type and size of snow grains were measured and manually classified according to the International Classification of Seasonal Ground Snow [5]. The disaggregated grains were placed on a grid millimeter map and observed with a 10× magnifying glass. The density of each layer was calculated using its mass and volume relative to the surface area of the core sampler (Table 3). In addition to the information collected on the physical parameters shown, a photo of each layer was visually associated to a general group of density, grain size, and type. The different shades of gray used in the bar graphs show the metamorphosis levels (low, moderate, high) of each recuperated snow layer. To ensure analysis coherence, all snow observations and measurements were made by the same individual.

**Figure 8:**
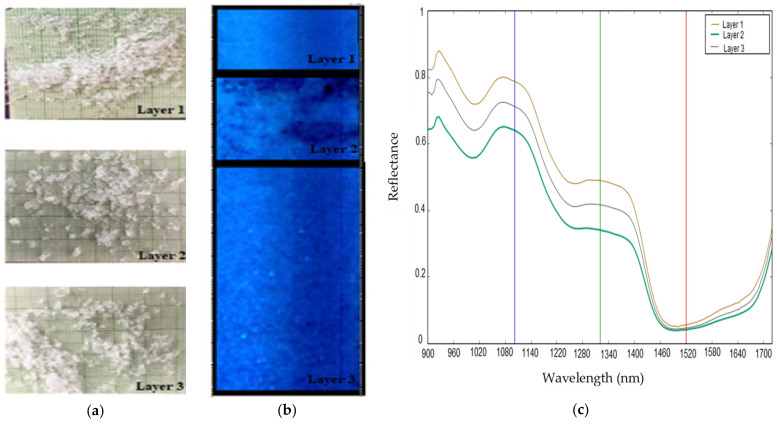
(**a**) Snow grains of each layer of the vertical stratigraphy; (**b**) false-color RGB image; (**c**) NIR spectral reflectance of each layer of the vertical stratigraphy (the red, green, and blue vertical lines in the spectral plot show the location of the bands used to generate the false-color RGB image).

**Figure 9:**
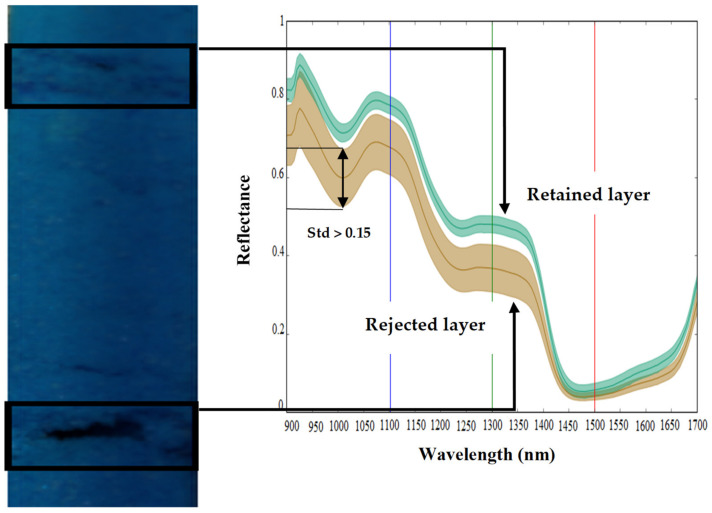
Snow layer retained (homogeneous), snow layer rejected (heterogeneous).

Based on the texture of the false-color RGB image (R = 1520 nm, G = 1320 nm, B = 1100 nm), it was possible to distinguish the different homogeneous layers of snow (Figure 8b). Layers estimated to be heterogeneous were removed from the initial database. The samples removed were those with a standard deviation (std) greater than 0.15 of the spectral reflectance, as presented in Figure 9. In total, five layers were removed. A high std reflects heterogeneity in the composition of a snow structure of the corresponding layer due to the presence of large air pockets which could lead to classification uncertainties. The mean spectral reflectance of each retained layer (Figure 8c) and the corresponding physical characteristics (Figure 8a) were measured. For a given layer of snow and between 900 nm and 1400 nm, the highest spectral reflectance values were generally observed for the largest physical characteristics (density, grain size and grain type) (Figure 8c).

**Figure 10:**
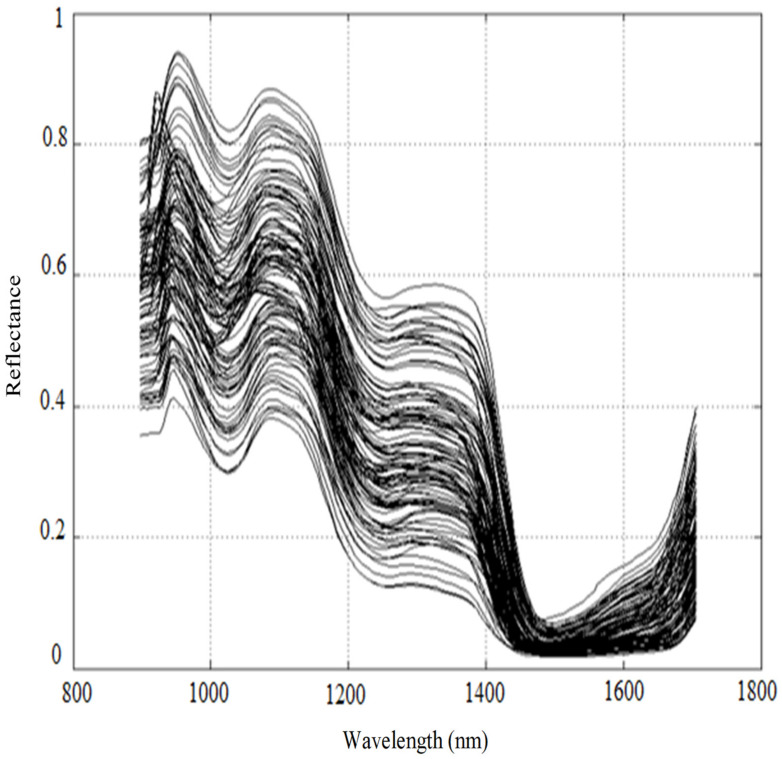
NIR spectral reflectance values of different homogeneous snow layers.

Figure 10 shows the spectral reflectance (114 samples) of the homogeneous layers of the snowpack’s vertical profile sampled in the winters of 2018, 2019, and 2020. It can be seen in Figure 10 that the spectra share the same shape but have different amplitudes (reflectance values). All spectra show a similar spectral behavior in the 1400–1700 nm bands, rendering band separation difficult. However, it is possible to detect a small difference between certain spectral bands, such as in the 1000–1100 nm and 1200–1300 nm bands, in which different groups of snow with close or homogeneous physical characteristics can be distinguished.

These spectral reflectance values show that the reflectance changes throughout the wavelength regions as snow ages [6,33]. Indeed, snow samples that were newly deposited on the snowpack (precipitation particles, decomposing, and fragmented precipitation particles) and that had a low to medium density (50–200 kg m^−3^) had higher reflectance values. On the other hand, samples of metamorphosed snow (goblets and melt forms) had lower reflectance values and a very high density (400–600 kg m^−3^). Samples of medium to high density (150–450 kg m^−3^) and moderately metamorphosed (rounded grains and faceted crystals) had intermediate reflectance values.

### 3.2. Visualization and Reduction of Spectral Dimensionality by Principal Components Analysis

**Figure 11:**
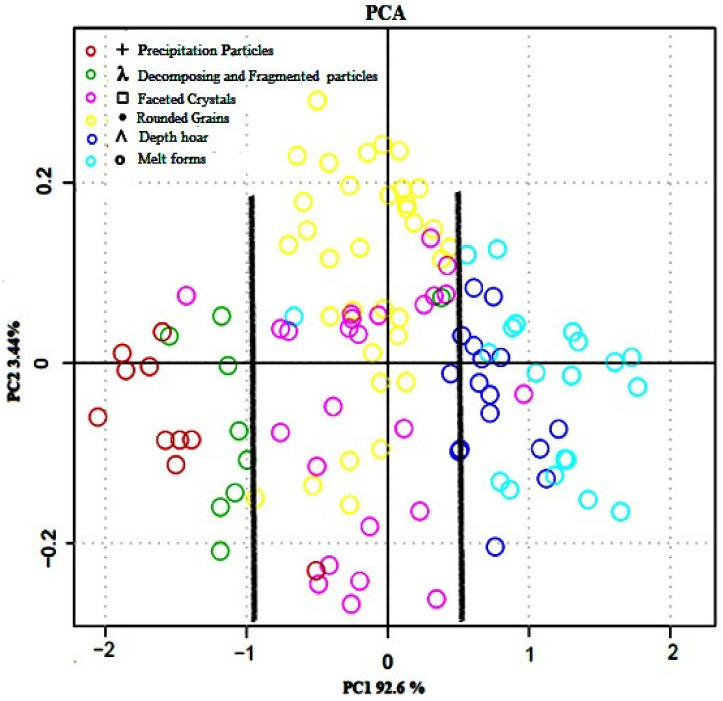
Score plot for PC1 and PC2 of spectral reflectance values. The cut-off bold lines help to visually distinguish among the snow classes in terms of metamorphosis degree (weakly, moderately, strongly metamorphosed snow).

A PCA was carried out on all spectral data. The first two principal components (PC) obtained were used to generate a score diagram, as they represented more than 96% of the variance of the spectral data. Figure 11 shows the score plot for PC1 and PC2, which seem to provide the best distinction between homogeneous snow groups in terms of grain type and size. Initially, the PCA was used to reduce the information recorded by the 148 spectral bands into two components so that it would be possible to project them onto a 2D score plot. Six snow groups can be distinguished (colored dots in Figure 11). None of these snow classes or snow groups can be considered atypical or aberrant.

The physical properties of snow of the same species were grouped together in each region of the PC1–PC2 space. Differences between most classes were pronounced and some snow types were mixed, such as rounded grains and faceted grains, which have not been clearly separated. This could be related to a similar grain size or shape. In short, PC1 and PC2 provide a good picture of the discrimination for the different physical characteristics of each snowpack. This implies that it is possible to optically classify snow layers based on their physical properties. Therefore, the score plot has the advantage of displaying information on sample groupings based on their physical characteristics and the degree of transformation or metamorphosis undergone by the snowpack.

Great similarities exist between the visualization of spectral data by PCA (optical data) (Figure 11) and the snow metamorphosis cycle formerly presented by Pahaut [15]. The origin of these snow groupings based on physical properties lie in the metamorphic processes undergone by snow grains after being deposited on top and incorporated into the snowpack, which continuously influence the snow’s spectral response. These processes operate in different ways depending on temperature, temperature gradient, and the amount of water in the snow [15]. Like other natural systems, the snowpack not only displays extreme examples of the phenomenon, but also intermediate stages and conditions of metamorphism. Metamorphism is the dominant factor in the physical evolution of the snow cover and it affects its stability (e.g., avalanche) and chemical composition. Consequently, detailed studies of the reflectance related to each physical attribute could lead to an important revolution in optical measurements.

**Figure 12:**
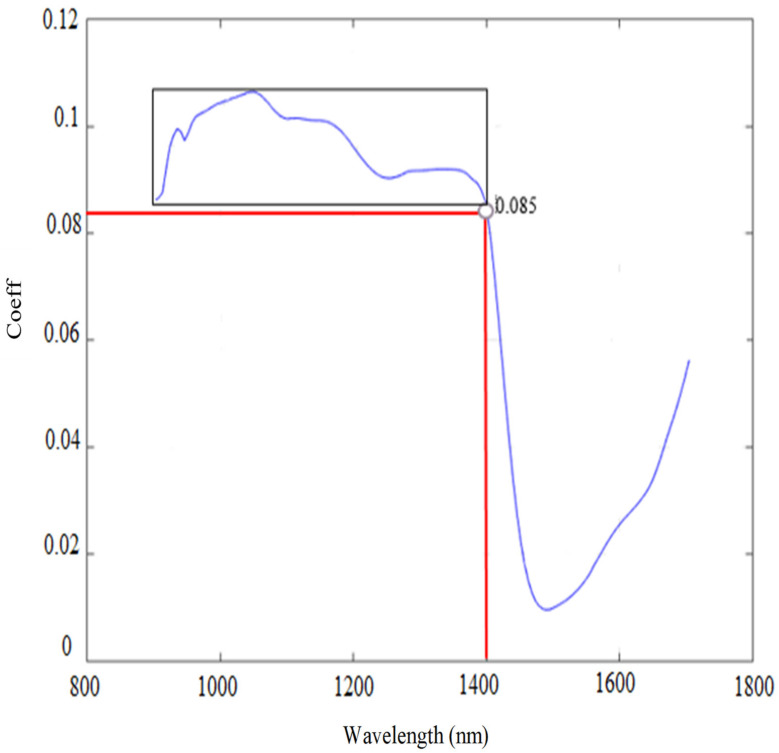
The coefficients of PC1 as a function of wavelengths.

The coefficients (Coeff) of the first PCA component were afterwards used to reduce the number of spectral bands on which the unsupervised classification model (HAC) was based; only bands with a Coeff greater than 0.085 were kept (above the red line from 900 to 1400 nm in Figure 12). HAC-based results were thereafter challenged using the corresponding field data for assessing the classification accuracy using the confusion matrix technique.

### 3.3. Unsupervised Spectral Reflectance Classification

According to the results of the PCA (Figure 11), it was found that spectral reflectance values can be preferably grouped into three distinct groups according to their spectral properties that reflect their physical compositions. The cut-offs (bold lines) were established between lightly metamorphosed snow (PP and DF), moderately metamorphosed snow (RG and FC), and strongly metamorphosed snow (DH and MF) (Figure 11). Admittedly, some misclassification remains between the spectra of certain classes, but this remains limited. This result is explained by the fact that the physical properties of these two groups of snow have undergone a very similar degree of metamorphosis, resulting in similar grain sizes and shapes.

**Figure 13:**
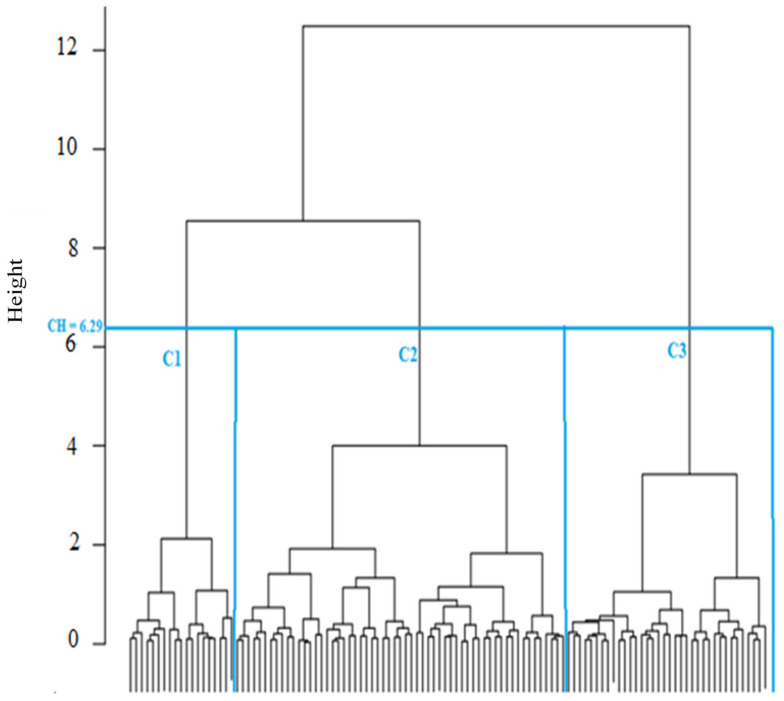
Dendrogram obtained using a hierarchical ascending classification; CH—cut-off height; C1—weakly metamorphosed, C2—moderately metamorphosed, and C3—strongly metamorphosed.

The classification was carried out using all the wavelengths constituting the spectral range from 900 nm to 1400 nm in order to focus the classification only on those bands that are most sensitive to changes in the size and type of snow grains undergone by the different types of metamorphism. The classification is ascending and hierarchical, as it produces increasingly larger classes or groups of snow, including subgroups (Figure 13). According to the resulting dendrogram, the snow classes have been separated at a relative distance of 6.29 (cut-off height (CH)), which produces the desired partition of three snow classes, corresponding to three degrees of snow metamorphosis (Figure 13).

**Figure 14:**
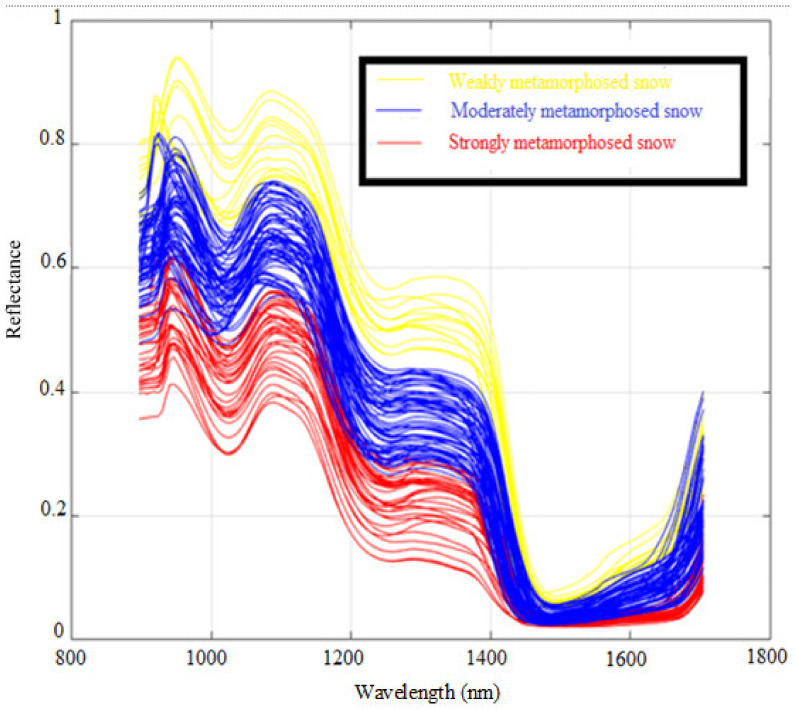
The near-infrared spectral reflectance values of the three snow classes.

**Figure 15:**
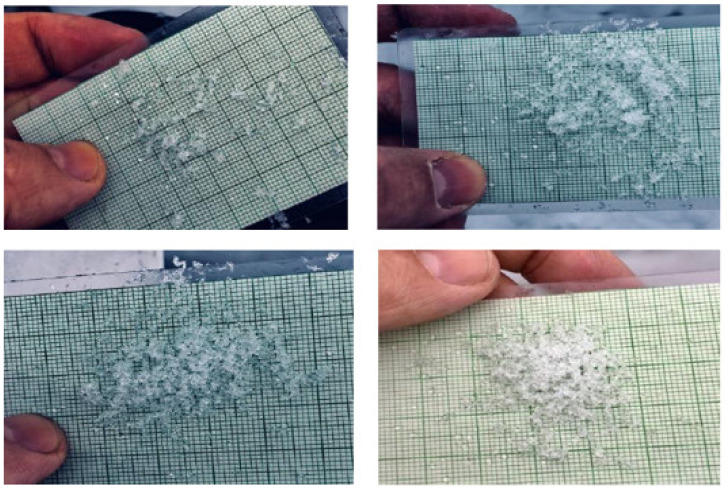
Snow grains from the weakly metamorphosed class.

**Figure 16:**
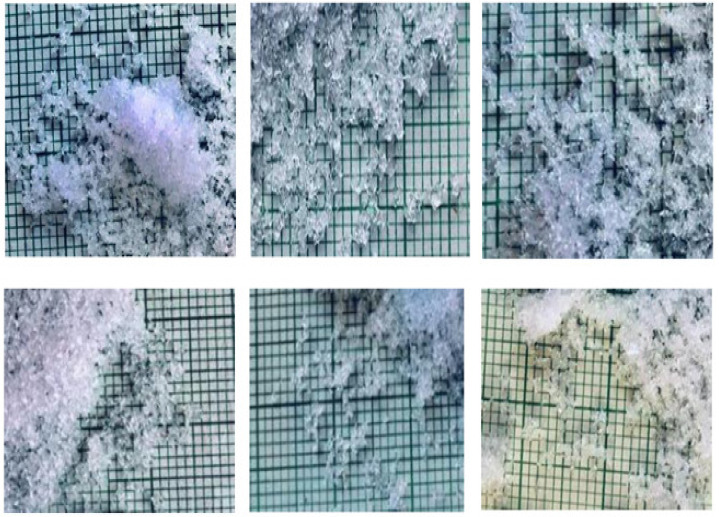
Snow grains from the moderately metamorphosed class.

**Figure 17:**
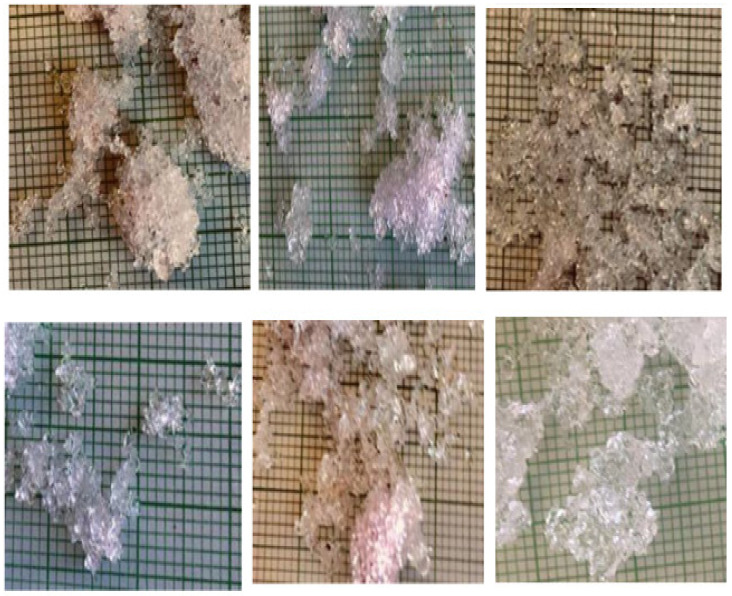
Snow grains from the strongly metamorphosed class.

Figure 14 shows the reflectance spectra of snow samples with different grain size and density ranges after the HAC. The three snow classes show separability in the 1000–1050 nm, 1150–1200, and 1350–1400 band ranges. As a result, the unsupervised HAC classification confirmed the results of the PCA (Figure 11), highlighting the spectral ranges where the three snow classes are most efficiently separated. The effect of the physical properties of snow on spectral reflectance can be explained by the negative relationship existing between the physical data and the optical properties of snow. This can be useful for the development of a model estimating the physical parameters of snow, such as the density. The result of the HAC classification led to a good differentiation between snowpack classes based only on optical data. By comparing Figure 15, Figure 16, Figure 17, which respectively correspond to weakly, moderately, and strongly metamorphosed snow, a morphological evolution of snow grain shapes and sizes can be observed. The classification method used is based on the continuous evolution of the physical characteristics of snow, and thus on the consequences of certain metamorphosis processes (destructive or constructive) [15], which place greater emphasis on the causes that provoke these different grain shapes (gradient or isothermal metamorphoses). Snow on the ground is not inert over time. From the moment it is deposited, it is subject to the combined effects of several factors, the most decisive of which are meteorological conditions [53]. Snow grains evolve after being deposited, and snow crystals change from their original form (fresh or recent snow) to granular forms (evolved snow or old snow). This sequence of continuous transformation and metamorphosis continues until the snow disappears during melting.

### 3.4. Method Assessement Using a Confusion Matrix

Classification performance was evaluated using a confusion matrix based on field data and corresponding optical data. Table 4 presents the results of the confusion matrix for the snow classes. The moderately metamorphosed snow class obtained the best result, followed by the strongly metamorphosed snow class and the weakly metamorphosed snow class, with respective commission errors of 10%, 19%, and 21%. Commission and omission errors were significantly higher for the weakly metamorphosed class than for the moderately and strongly metamorphosed classes. Misclassification usually occurs at the extreme of each class (in the weakly–moderately and moderately–strongly class areas). In other words, the error due to in situ data sampling introduced various levels of uncertainty into the field database, which has inevitably affected the precision. These uncertainties are related to the sampling method used, especially when recovering layers of weakly metamorphosed snow. These are the most likely to have affected the accuracy of the values in the measured database, and therefore affect the ability to correctly classify snow samples. Despite the above limitations, the performance of the classification was very acceptable (kappa index = 0.75 and overall success = 0.85%).

## 4. Conclusions

In this work, a hyperspectral imaging system in the NIR spectrum was used to discriminate and classify the snow layers of the vertical profile of the seasonal snowpack according to their physical properties. An unsupervised classification (HAC) analysis was performed based on the reflectance spectra of different snow varieties. The confusion matrix indicated acceptable performance of the data classification, with a global success of 85%. This showcases the potential of NIR hyperspectral imagery for selecting snow classes based on their spectral data. The proposed approach has proven to be an appropriate and effective method for distinguishing snow classes.

The results highlight the fact that hyperspectral NIR imaging between 900 nm and 1400 nm could be used to classify snow samples, even without any prior knowledge of physical properties. Multivariate statistical analysis techniques such as PCA and HAC have the advantages of being objective, rapid, and non-destructive techniques for the authentication and classification of snow on the ground. The distinctive spectral difference between snow grades could be explained in various wavelengths in the NIR range of the spectrum, since they are related to water and other physical and chemical components. However, we recommend that this method be tested on a wide variety of sites presenting a wide range of snowpacks (low vs. high altitude, close vs. open environments, coastal vs. continental conditions, etc.) in order validate its robustness further, and if necessary, recalibrate it using these additional observations for better accuracy.

The main limitations associated with this method are the smaller number of layers in the weakly metamorphosed class, as well as homogeneous snow layer identification on the acquired hyperspectral image. The first constraint is due to the difficulty of isolating this type of layer, because weakly metamorphosed snow is very light and often thin. The second constraint could be overcome by validating the homogeneous snow layers on a field sampler before starting the optical processing. Additional studies are needed in this direction to eradicate these drawbacks and eventually lead to field implementation. In general, the results demonstrated by recent works have shown that this technology meets the scientific need for a fast and accurate classification method for discriminating snow samples on the ground, and furthermore, to estimate the physical characteristics of snow.

The contribution of this work is to present a rapid and non-destructive approach to discriminate between different types of snow on the ground that could be used in the near future. At present, there is no method that uses optical analysis for discriminating these varieties. The procedure proposed herein opens the window for the subsequent introduction of cheaper multi-spectral or hyperspectral NIR instruments that could be used for the desired application.

## Figures and Tables

**Table 1:** Temperature data report for the winters of 2018, 2019, and 2020.

Winter Season	Temperature (°C)							
	January		February		March		April	
	Max	Min	Max	Min	Max	Min	Max	Min
2018	−7.7	−18.3	−2.1	−15.2	1.6	−7.1	5.5	−4
2019	−8.2	−19	−6.2	−18.7	−0.2	−12.7	6.2	−1.6
2020	−4.5	−13.2	−3.2	−17.1	1.8	−8.6	7.1	−2.8

**Table 2:** Ground snow accumulation data report for the winters of 2018, 2019, and 2020.

Winter Season	Snow on the Ground (cm)							
	January		February		March		April	
	Max	Min	Max	Min	Max	Min	Max	Min
2018	67	24	81	58	80	48	56	2
2019	79	35	105	64	105	70	71	7
2020	49	14	76	47	85	61	64	0

**Table 3:** Distribution of snow density in terms of type and size of snow grains (field data: 2018, 2019 and 2020), with their corresponding symbols. PP—precipitation particles; DF—decomposing and fragmented precipitation particles; RG—rounded grains; FC—faceted crystals; DH—depth hoar (goblets); MF—melt forms. The different shades of gray show the levels of metamorphosis (low, moderate, high) of each recuperated snow layer.

Grain Type	Grain Size	Number of Samples	Density (kg m^−3^)																	
			<100	100–150		150–200			200–250	250–300		300–350	350–400			400–450	450–500		>500	
+ (PP)	<1 mm	9	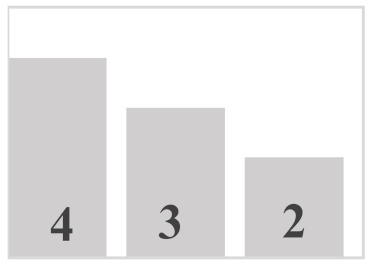																	
λ (DF)	<1 mm	10			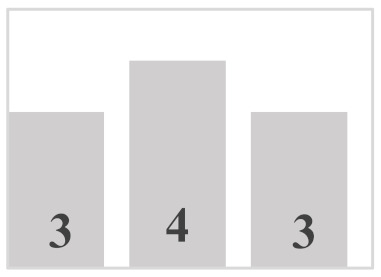															
□ (FC)	1–2 mm	25					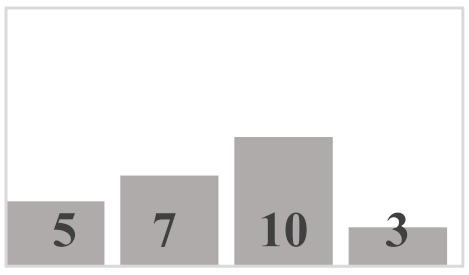													
• (RG)	1–2 mm	35						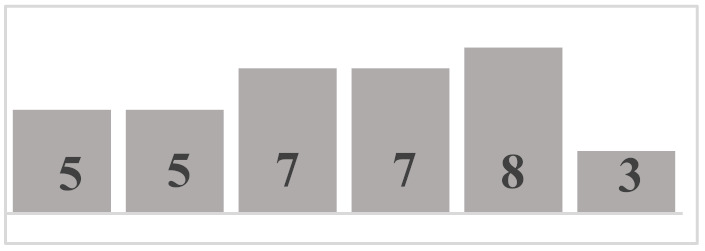												
˄ (DH)	>2 mm	16													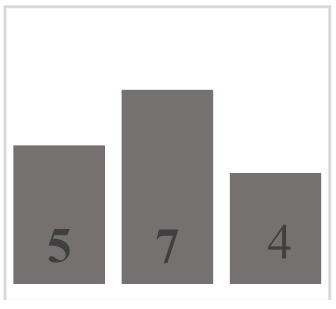					
ᴼ (MF)	>2 mm	20													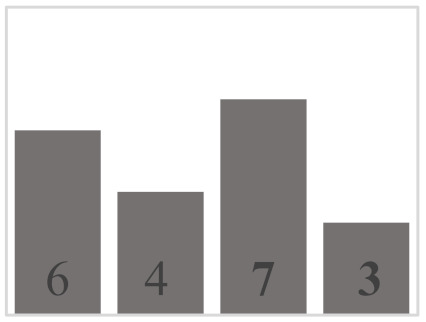					

**Table 4:** Confusion matrix results; C1—weakly metamorphosed, C2—moderately metamorphosed, C3—strongly metamorphosed.

Observed							
**Estimated**		C1	C2	C3	Total	Commission error (%)	Success rate (%)
	C1	15	3	1	19	21%	83
	C2	3	52	3	58	10%	90
	C3	3	4	30	37	19%	81
	Total	21	59	34	114	-	-
	Error omission (%)	29	12	12	-	-	-
	Success rate (%)	71	88	88	-	-	-
	Global success (%)	-	-	-	-	-	0.85
	Kappa Index	-	-	-	-	-	0.75

## Data Availability

Not applicable.
